# How Multidisciplinary Teams Are Reshaping Adult IBD Management: A Narrative Review

**DOI:** 10.3390/jpm16100536

**Published:** 2026-10-21

**Authors:** Alessia Todeschini, Maria Vittoria Teso, Giuliano Barugola, Angela Variola

**Affiliations:** 1 Inflammatory Bowel Diseases Unit, IRCCS Sacro Cuore Don Calabria Hospital, 37024 Negrar di Valpolicella, Italy; angela.variola@sacrocuore.it; 2 Gastroenterology Unit, Department of Medicine, The Pancreas Institute, University Hospital of Verona, 37134 Verona, Italy; mariavittoria.teso@univr.it; 3 General Surgery Unit, IRCCS Sacro Cuore Don Calabria Hospital, 37024 Negrar di Valpolicella, Italy

**Keywords:** inflammatory bowel disease, multidisciplinary team, integrated care, care delivery models, IBD nurse

## Abstract

Current guidelines strongly endorse integrated multidisciplinary team (MDT) approaches to manage the complexity of patients with IBD. Evidence from formal multidisciplinary case discussions suggests an impact on diagnostic and treatment decisions, while studies of broader integrated IBD services report associations with improved disease control, quality of life, and healthcare utilization. However, implementation remains heterogeneous across centers, with variability in team composition, meeting frequency, referral criteria, and access to specialist services. Future directions include standardization of MDT composition and processes, definition of quality indicators, extension of specialist expertise through hub-and-spoke networks and telemedicine, and integration of patients as active partners in care. Digital tools may support communication and remote follow-up, whereas the role of artificial intelligence in MDT decision-making remains investigational and requires clinical validation. Multidisciplinary care should be regarded as a core organizational principle of modern IBD management. Realizing its full potential will require sustained investment, workforce planning, and prospective research to ensure equitable, high-quality, and sustainable care across diverse healthcare settings.

## 1. Introduction

Inflammatory bowel disease (IBD), comprising Crohn’s disease (CD) and ulcerative colitis (UC), is a group of chronic, relapsing, and systemic inflammatory disorders with a steadily increasing prevalence in industrialized countries and a rising incidence in emerging regions [1]. Although IBD is primarily characterized by gastrointestinal symptoms, its clinical course often requires chronic monitoring, repeated therapeutic adjustments and reassessment. However, the disease burden extends beyond the gastrointestinal tract, encompassing extraintestinal manifestations and psychosocial challenges that demand long-term multidisciplinary management.

In this context, single-specialty care models are frequently insufficient to address the complex needs of patients with IBD, making holistic multidisciplinary care an emerging key component in the comprehensive management of the disease burden.

## 2. Methods

This is a narrative review summarizing current evidence on multidisciplinary team (MDT) care in inflammatory bowel disease (IBD). We searched PubMed/MEDLINE, with the last search performed on 25 May 2026, using the following search strategy: (‘Inflammatory Bowel Diseases’[Mesh] OR ‘Crohn Disease’[Mesh] OR ‘Colitis, Ulcerative’[Mesh] OR IBD OR Crohn OR ‘ulcerative colitis’) AND (‘Adult’[Mesh] OR adult*) AND (‘Team-Based Care’[Mesh] OR ‘Multidisciplinary Approach’[Mesh] OR ‘Patient Care Team’[Mesh] OR ‘Transitional Care’[Mesh] OR multidisciplinary OR interdisciplinary OR ‘team-based care’ OR ‘multidisciplinar* team’ OR ‘multidisciplinar* care’ OR ‘multidisciplinar* management’). Filters were applied to include articles published in English between 2006 and 2026. Earlier seminal publications and authoritative background documents were also included when considered necessary to contextualize the development of integrated care models. We prioritized guidelines, consensus statements, systematic reviews, and original studies reporting on MDT composition, organizational models, clinical outcomes, economic impact, or implementation strategies. Articles were selected based on their relevance to the scope of the review, with no formal quality assessment or meta-analysis performed. This narrative approach was chosen to provide a comprehensive and clinically oriented synthesis of heterogeneous evidence, including observational studies, expert opinions, and real-world experiences that would not be captured by a strictly systematic methodology.

## 3. Rationale for Multidisciplinary Care in IBD

### 3.1. The Multi-Dimensional Nature of IBD

The management of IBD as a chronic systemic disease encompasses a broad spectrum of clinical manifestations and patient needs. Up to 50% of patients with IBD manifest at least one extraintestinal manifestation (EIM) [2]. EIMs may occur at any stage of the disease course, preceding the onset of IBD in up to 24% of patients, and are often associated with concomitant intestinal disease activity [3]. Immune-mediated EIMs most commonly affect the musculoskeletal, dermatologic, hepato-pancreato-biliary, and ocular systems; however, virtually any organ system may be involved. Moreover, the European Crohn’s and Colitis Organisation (ECCO) consensus guideline on EIMs broadens this concept by including disease- and treatment-related extraluminal complications, as well as associations with other immune-mediated disorders [4]. Disease phenotype also contributes substantially to the overall burden, particularly in patients with perianal CD, stricturing or penetrating behavior, and post-surgical complications such as stoma care, intestinal failure, and pouch-related disorders. Beyond organ-specific manifestations, patients with IBD frequently experience an impaired quality of life (QoL), reduced social and occupational functioning, and psychosocial distress that frequently require dedicated supportive interventions [5,6,7]. In addition, the aging IBD population needs further consideration of coexisting multimorbidity, polypharmacy, and frailty. On the other hand, children and adolescents transitioning to adult care necessitate a guided and timely transfer process, involving both their families and the multidisciplinary specialists [8]. In this multifaceted clinical landscape, IBD management is moving beyond a solely gastroenterology-reliant approach towards a multidimensional and multispecialistic assessment truly incorporating personalized and integrated care pathways.

### 3.2. Why Multidisciplinary Care Is Needed in IBD

Current guidelines and quality standards strongly recommend the adoption of multidisciplinary team (MDT) care across specific gastroenterological settings. The MDT model is well established in complex fields such as gastrointestinal oncology, where the shared decision-making process has consistently been associated with improved clinical outcomes [9,10]. The World Health Organization has advocated integrated, coordinated models of care for chronic conditions, an approach that has subsequently been adopted and adapted to IBD management [11].

Current ECCO guidelines recommend multidisciplinary care and collaborative decision-making as key components of patient-centered management, encompassing pharmacological, surgical, nutritional, and other non-pharmacological interventions [12,13].

Compared with traditional single-specialty care, multidisciplinary management and integrated IBD services have been associated with improved clinical outcomes, including fewer hospitalizations, reduced loss of response to therapies, lower rates of IBD-related surgery, changes in healthcare utilization, and improved patient education and QoL [14,15,16]. Despite its recognition as a standard of care, implementation remains highly variable across centers. Differences in healthcare organization, limited access to dedicated professionals and resource constraints continue to represent major barriers to equitable MDT care across centers. For this reason, the development of the broadest feasible multidisciplinary network remains a key organizational priority in modern IBD care [17].

## 4. Multidisciplinary Team Composition

The holistic and personalized management of patients with IBD requires a synergistic collaboration among various healthcare professionals to achieve consensus and standardization of care [14,18]. Nevertheless, strong evidence defining the ideal composition and structure of the IBD MDT remains limited. MDT members are typically categorized into core or extended team members based on the frequency of their involvement, the nature of their responsibilities, and the clinical objectives pursued.

### 4.1. Core Team Members

The core team generally includes a gastroenterologist, a colorectal surgeon with specific expertise in IBD and an IBD nurse who may also serve as coordinator. The second N-ECCO consensus distinguishes *fundamental IBD nursing*, which requires core disease-specific knowledge, from *advanced IBD nursing*, which involves specialist expertise and may be supported by postgraduate education [19]. Endoscopists and radiologists contribute to diagnostic assessment and disease monitoring; in a consensus led by Louis et al. [20], 81% of respondents agreed that IBD units should have access to a dedicated endoscopist and to cross-sectional imaging integrated into routine care.

Dietitians, stoma nurses, and psychologists are also frequently included as core members to address malnutrition, stoma-related issues, and psychosocial needs. Dietitians can assess nutritional risk, guide dietary intervention, and support patients before and after surgery [21]. Nevertheless, some MDTs do not routinely include a dietitian, possibly due to limited availability of dietetic services or a lack of awareness regarding their contribution [22]. Psychologists may assess coping, treatment-related distress, sexuality and the effects of surgery or stoma formation and support patients at key stages such as diagnosis, flares and transition to adult care [23,24]. While most teams comprise gastroenterologists, surgeons, nurses and dietitians, mental-health practitioners are less commonly represented, although they are considered essential [25]. Social workers may complement psychological care by identifying practical and socioeconomic barriers to treatment and connecting patients and families with financial assistance, employment or school accommodations, and community resources [26].

In integrated models, pharmacists may contribute to medication reconciliation and optimization, adherence support, interaction and safety review, and patient education [27]. Gastrointestinal pathologists play a fundamental role in the diagnosis of IBD and in distinguishing it from other forms of colitis or enteritis many studies include them only as extended members of the MDT or even as ad hoc participants [24]. An American retrospective study reported that up to 42% of patients had a change in diagnosis after MDT discussion following expert histopathologic review [28].

### 4.2. Extended Team Members

**Figure 1:**
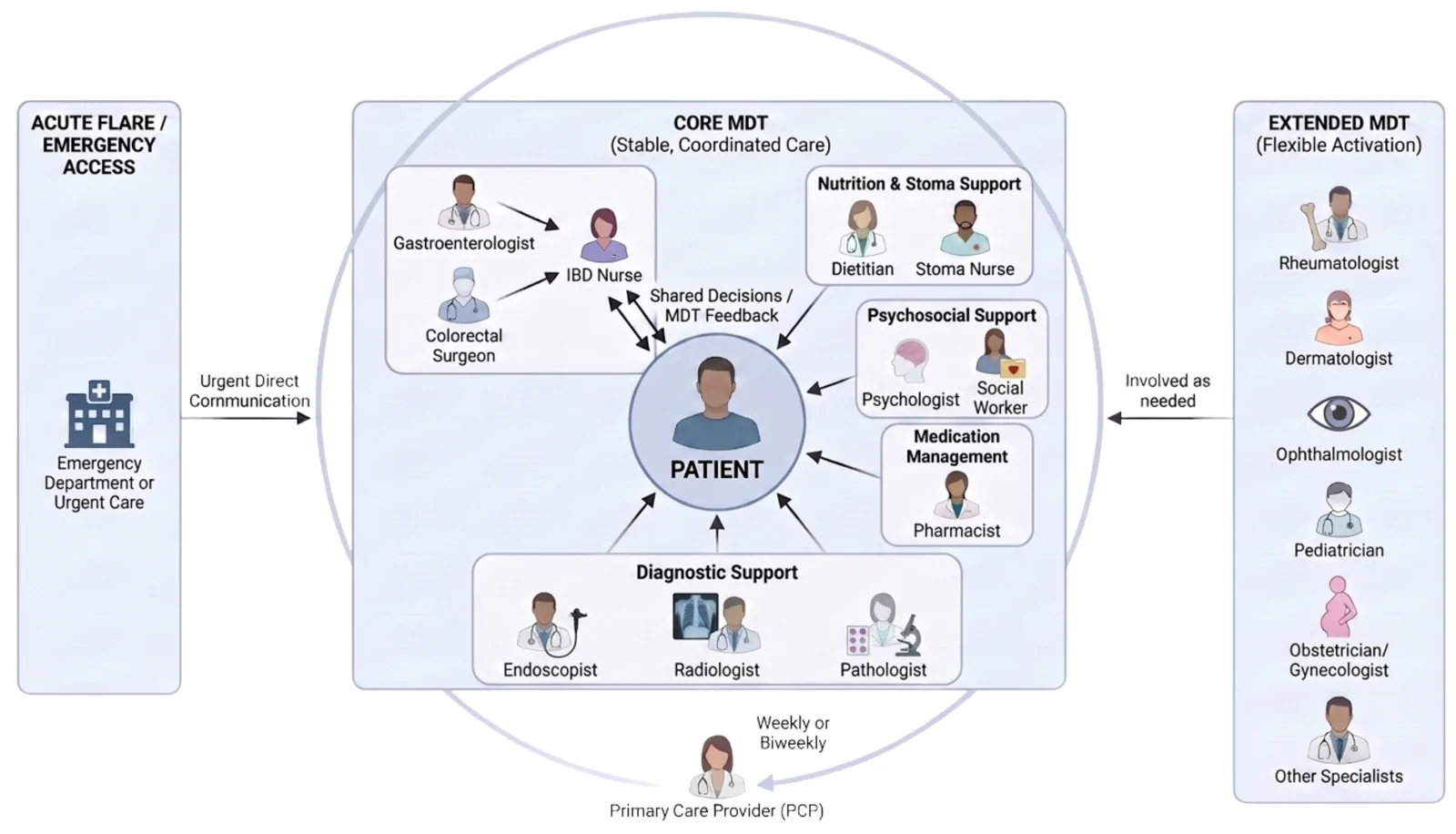
Multidisciplinary Team Composition. Abbreviations: IBD = Inflammatory Bowel Disease; MDT = Multidisciplinary Team; PCP = Primary Care Provider. Created in BioRender. Teso, M. V. (2026) https://BioRender.com/o9b2ihl.

The extended or ad hoc team includes specialists involved based on individual patient needs or the presence of EIMs, such as rheumatologists, dermatologists, and ophthalmologists. Pediatricians are essential for managing the transition from pediatric to adult clinics, while obstetricians and gynecologists provide specialized care for pregnancy and fertility planning. The composition of the extended network may vary according to local resources, hospital characteristics, and patients’ needs [29]. Ad hoc members are involved in a more targeted manner, collaborating only for specific patients or clinical situations in which the complexity of the case requires ultra-specialized expertise that is not available within the core group. Their participation is typically based on invitations from core members and may be activated when particular diagnostic or therapeutic concerns arise or when complications occur. Together, the core and ad hoc components of the team allow the service to combine stability and continuity with flexibility and access to highly specialized expertise when needed (Figure 1) [30].

## 5. Models of Multidisciplinary Care Delivery

This review distinguishes formal multidisciplinary team meetings (MDMs), focused on discussion and management of individual cases, from broader models of integrated care. Dedicated IBD services, specialty medical homes, nurse- and pharmacist-led pathways, joint specialty clinics, transition programs, flare clinics, and perioperative pathways may involve multidisciplinary collaboration but also modify staffing, access, monitoring, and care coordination. Outcomes from these models are therefore attributed to the service or pathway evaluated, not to an MDT meeting in isolation.

### 5.1. Multidisciplinary Team Meetings

Although multidisciplinary IBD care is widely recommended, the optimal composition, working procedures, and implementation of multidisciplinary teams remain poorly standardized across centers [12,16,17]. Current recommendations suggest that MDMs should be supported by a dedicated coordinator responsible for meeting organization, database management and communication among team members. The frequency of MDT meetings should reflect the volume of patients requiring discussion. Ideally, meetings should be held on a weekly or biweekly basis to ensure that decision-making is not delayed. Acute or emergency decisions should be addressed separately through direct communication between involved physicians [18]. All team members should be encouraged to attend the meetings; non-attendance, particularly among core members, represents a barrier to effective shared decision-making [30].

Effective communication and collaboration among healthcare providers are central to the MDT care pathway [20]. For each case, the agreed actions, timeframe, and clinician responsible for implementation should be specified. MDT decisions should be documented and subsequently discussed with the patient [31]. Additionally, communication between the IBD team and the patient’s primary care provider (PCP) is also an important component of outpatient IBD care. Although the PCP is not a formal member of the IBD team, they can support patient education, encourage adherence, and assist in delivering preventive care, including maintaining up-to-date vaccinations [15].

Appropriate patient selection is fundamental to maximize the value of discussion; however, no consensus criteria currently exist regarding which patients should be referred [32]. In practice, priority may be given to diagnostic uncertainty, complex medical or surgical decisions, and cases requiring input from several specialties. Referrals should identify the clinical question and provide relevant treatment history, laboratory results, and endoscopic, imaging, or histopathological findings, as applicable. Attendance, time from case submission to discussion, and completion of agreed actions are potential measures for auditing the MDT process. While multidisciplinary discussion of the patient is considered fundamental to high-quality IBD care, its implementation remains inconsistent. Across Europe, the recent ECCO E-QUALITY survey found that regular MDMs were available in only 67% of IBD units, despite most centers reporting an interdisciplinary approach [33].

### 5.2. Nurse-Led Practice

Nurses play an important role in the care of patients with IBD across all healthcare settings. Advanced IBD nurses are considered a key component of IBD MDTs, facilitating communication between patients and the team while providing holistic, patient-centered care, including clinical assessment, patient education, care coordination, and monitoring of biologic therapies. Their role also extends to research, education, service development, and leadership within IBD services [19,34]. Moreover, they can ensure accessibility to care by providing rapid-access and remote services, allowing more flexible management while facilitating early assessment of disease flares and reducing unnecessary outpatient visits and emergency attendances through timely referral to the most appropriate level of care [16,19,35,36,37]. In a prospective study of newly diagnosed patients with CD offered structured MDT management, 90% proactively communicated with the IBD nurse via email, messages, phone calls, or nurse visits between gastroenterological assessments, suggesting that flexible communication channels are highly valued and facilitate continuous patient engagement [38].

### 5.3. Integrated Multidisciplinary Outpatient Clinics

More recently, the United States has introduced the IBD Specialty Medical Home (SMH), an innovative care model in which a dedicated MDT coordinates longitudinal disease management, acute care, and preventive interventions to improve continuity and reduce unplanned healthcare utilization [31]. Implementation of the IBD SMH has been associated with a 47.3% reduction in emergency department visits, a 35.9% reduction in hospitalizations, significant improvements in disease activity, and better QoL after one year of follow-up [39]. Other models of integrated outpatient care include dedicated flare clinics (FCs). Initially developed during the COVID-19 pandemic, the Edinburgh group evaluated that FCs identified patients requiring hospital admission as an alternative to conventional emergency gastroenterology services [40]. These integrated outpatient models are particularly valuable because they combine specialist oversight with flexible, patient-centered access pathways, allowing early intervention during flares and more efficient use of MDT resources.

## 6. Clinical Outcomes and Healthcare Economic Impact

### 6.1. Disease Activity and Remission Rates

**Figure 2:**
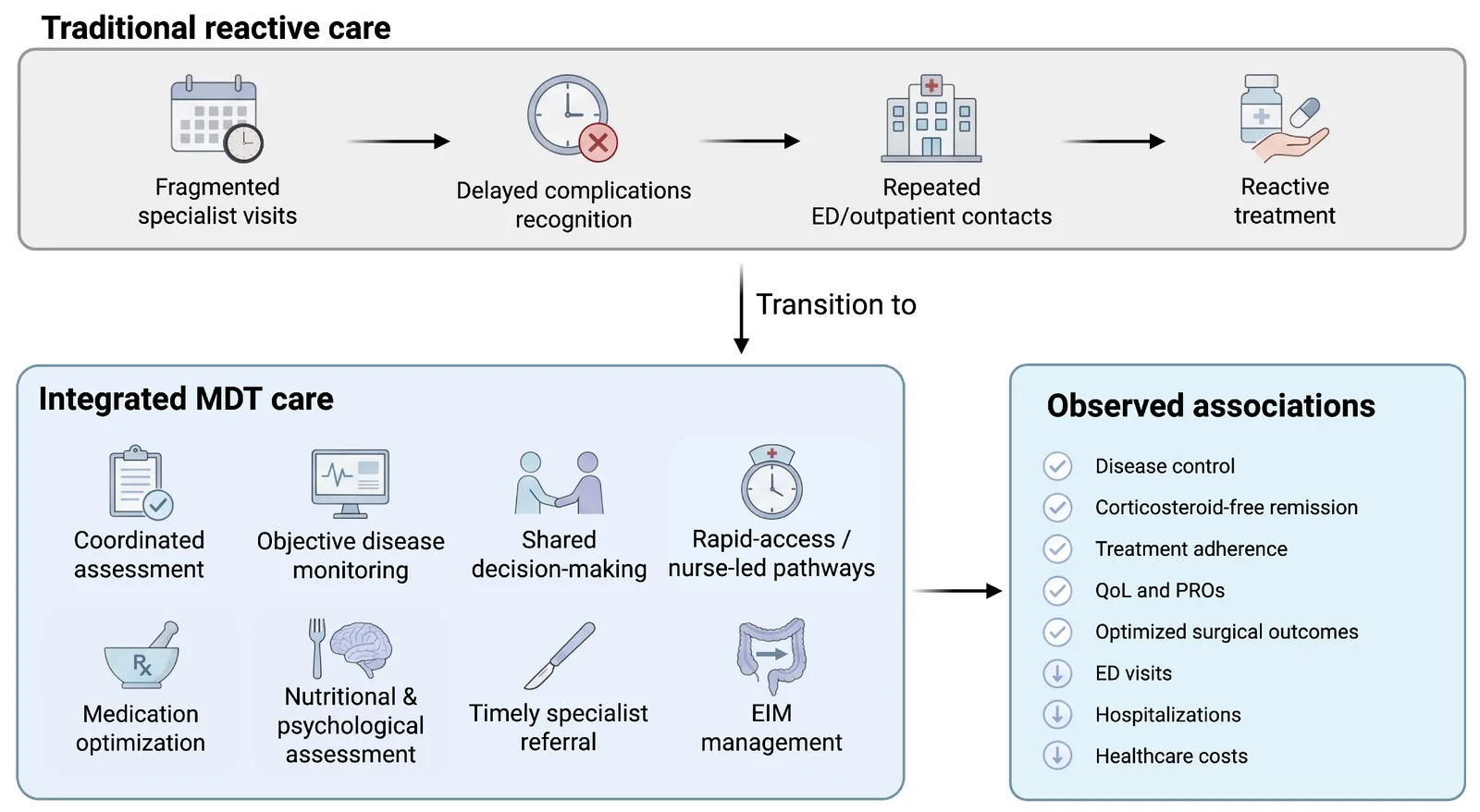
From fragmented to integrated IBD care: coordinated, proactive, and patient-centered care has been associated with improved clinical and patient-reported outcomes and reduced healthcare utilization. The outcomes shown represent reported or observed associations, primarily from observational studies of heterogeneous integrated-care interventions. Abbreviations: EIM = Extraintestinal Manifestation; ED = Emergency Department; MDT = Multidisciplinary Team; PRO = Patient-Reported Outcome; QoL = Quality of Life. Created in BioRender. Teso, M. V. (2026) https://BioRender.com/wgtay0x.

The available evidence suggests that structured multidisciplinary care may be particularly beneficial when it combines objective disease monitoring, early therapeutic intervention, and coordinated management of complex disease phenotypes (Figure 2). However, the evidence derives from heterogeneous interventions, including dedicated IBD services, MDT meetings, and specialized clinics.

In a prospective study on newly diagnosed patients with CD managed with a structured MDT approach, 64.5% of patients achieved sustained corticosteroid-free clinical remission (CS-free CR) at 1 year, 44.2% achieved endoscopic remission and 30.8% deep remission [38]. Although these outcomes appear favorable compared with those reported in several registration trials, direct comparisons should be interpreted cautiously because of differences in study populations, treatment protocols, definitions of remission, and follow-up duration. A comparative study of dedicated MDT hasreported significant reductions in clinical recurrence rates compared with pre-implementation cohorts (*p* < 0.005). In patients with CD, successful implementation of MDT decisions was associated with clinical remission in 58.2% of patients after 27 months, compared with 15.4% when recommendations were not implemented within 6 months [41]. These observational findings suggest that the implementation of MDT recommendations depends not only on the quality of shared decision-making but also on the timely implementation and subsequent monitoring of its recommendations.

Multidisciplinary collaboration is particularly valuable in patients with concomitant immune-mediated inflammatory diseases (IMIDs). In a gastro-rheumatology clinic, combined intestinal and articular remission progressively increased from 0.6% at baseline to 26.3% after four years [42]. In another cohort, multidisciplinary management was associated with sustained improvements in overall health-related QoL, encompassing both gastrointestinal and rheumatological outcomes at 12 and 24 months, with 72.5% of patients initiating biologic disease-modifying antirheumatic drugs [43]. In one study, 57% of rheumatological-gastrointestinal MDMs were followed by an immediate change in disease management [44]. In addition, they facilitated the development of a therapeutic strategy that avoided the need for dual therapy, limiting unnecessary treatment escalation.

The benefits of multidisciplinary care observed in adults are also supported by evidence in the pediatric and adolescent setting. Although pediatric care is outside the primary scope of this review, transition programs are relevant because they support transfer into adult IBD services and aim to prevent gaps in follow-up. Structured transition programs are intended to promote self-management skills, prevent gaps in follow-up, and may improve treatment adherence [45,46]. Planned transition has also been associated with higher clinical remission one year after transfer than those who do not receive structured transition care (18 out of 21 vs. 11 out of 24 patients, respectively; *p* = 0.037) [47]. Overall, these findings support a positive association between multidisciplinary care and disease control across different IBD populations. However, the available evidence remains largely observational and heterogeneous. The observed benefits may reflect the combined effects of specialist expertise, improved access, nurse-led follow-up, structured monitoring, patient engagement, and more timely implementation of treatment decisions, rather than the isolated effect of the MDT itself.

### 6.2. Medication Optimization, Adherence and Corticosteroid-Free Remission

Structured MDT pathways can facilitate timely treatment adjustments through coordinated assessment of disease activity, treatment history, safety parameters, adherence, and patient preferences. However, evidence derives from different models, including comprehensive MDT services and pharmacist-led interventions.

In a prospective cohort of newly diagnosed patients with CD managed within a structured MDT pathway, 64.5% achieved sustained CS-free CR at 1 year [38]. Although these findings are consistent with a potential contribution of structured multidisciplinary care to treatment engagement and steroid-sparing outcomes, the study was not designed to establish a direct causal relationship between adherence and remission. A survey following the implementation of a formal multidisciplinary IBD service observed significant reductions in corticosteroid and opioid use and overall medication burden (all *p* < 0.05) [48]. Following implementation of a pharmacist referral service, referral rates rose from 48% to 72%, accompanied by improvements in disease activity, while the proportion of patients not using steroids increased from 50% to 63% [49]. In a prospective study, pharmacist-led medication reviews resulted in the initiation of new therapies in 35% of patients and generated interventions involving 79% of all prescribed medications [50]. Importantly, 92% of the pharmacist recommendations were accepted and implemented by clinicians within the MDT [50]. Overall, multidisciplinary care may promote medication optimization and corticosteroid-free remission through several complementary mechanisms: earlier recognition of inadequate treatment response, improved adherence support, coordinated safety monitoring, pharmacist involvement, and greater patient participation in therapeutic decisions. However, the available evidence remains mainly observational, and further prospective studies are needed to establish which MDT components most effectively improve adherence and long-term steroid-free outcomes.

### 6.3. Surgical Outcomes

Although high-quality comparative evidence remains limited, an integrated medical-surgical approach is increasingly regarded as the standard of care for patients with complex IBD requiring surgery, particularly those with penetrating CD, complex perianal disease, or medically refractory UC [51,52]. In a single-center retrospective study of patients with IBD undergoing surgery, clinical recurrence and the need for additional surgery were less frequent among patients discussed by an MDT than among those operated on before MDT implementation (*p* < 0.005 and *p* < 0.01, respectively) [53]. In a study of Perioperative Surgical Homes, integrating preoperative assessment, nutritional and medical optimization, coordinated perioperative management, and postoperative rehabilitation has demonstrated a significantly lower incidence of complications than the standard of care (26.4% vs. 44.9%, *p* = 0.035), alongside a shorter hospital stay [54]. These observations suggest that the benefit of multidisciplinary surgical care may result not only from the operative strategy itself but also from better patient selection, optimization before surgery, and continuity of care after discharge.

The role of an MDT is particularly important in perianal fistulizing CD, one of the most complex and disabling disease phenotypes. In a prospective single-center study incorporating specific patient-reported outcome measures (PROMs) into treatment decisions, definitive surgery was achieved in more than half of patients without compromising continence, while QoL improved significantly. However, clinical recurrence occurred in 31.6% of patients during a median follow-up of 14.5 months [55]. These findings highlight the importance of evaluating not only fistula healing but also continence, symptom burden, functional outcomes, and patient quality of life. Similarly, a new protocolized three-stage MDT approach for perianal fistulizing CD resulted in higher rates of clinical healing (71%) compared to standard treatments, improved radiologic remission (25%), and a low relapse rate (7%) during a median follow-up of 23 months [56]. Although these results are promising, they derive from non-randomized studies and should be confirmed in larger multicenter cohorts using standardized definitions of clinical and radiological healing. The most robust evidence currently concerns complex perianal CD and perioperative pathways, whereas the impact of MDT care on overall surgery success rates and long-term postoperative recurrence remains less clearly established.

### 6.4. Patient-Reported Outcomes and Quality of Life

PROs are measures of patients’ health status, symptoms, functioning and QoL. They are increasingly recognized as important domains to assess treatment efficacy and are being incorporated more frequently into clinical trials [57] and routine IBD care [58]. PROs are particularly relevant in IBD because the burden of disease is not fully captured by inflammatory biomarkers, endoscopic findings, or clinical indices. In a population-based cohort of newly diagnosed patients with IBD, approximately 80–85% reported moderate-to-severe impairment in at least one PRO, with QoL being the most frequently affected domain, followed by fatigue and disability [59]. Disease-specific QoL scores, including the Short Inflammatory Bowel Disease Questionnaire (SIBDQ), improved from the initial MDT assessment throughout follow-up [39,60]. Among newly diagnosed patients with CD managed within an MDT, 39.5% reported improved health-related QoL after one year of follow-up [38]. These findings suggest that the benefits of coordinated care may extend beyond disease activity and include improvements in patients’ perception of health and daily functioning. Depression and anxiety are common in IBD and may negatively influence health-related QoL, treatment adherence, and engagement with healthcare services [61].

Dedicated IBD nurse support represents another important contributor to patient-centered outcomes. In the randomized IBD BIO-ASSIST trial, additional specialist IBD nurse support did not significantly improve disease-specific quality of life in the overall intention-to-treat analysis; a benefit at 18 months was observed only in a per-protocol analysis of patients with impaired quality of life at baseline [62]. A survey of patients attending an IBD clinic with specialized IBD nurses within an MDT demonstrated better disease-specific PROs, including higher self-efficacy scores, compared with those receiving standard care [63]. However, the evidence remains heterogeneous, and improvements in PROs may reflect the combined effects of disease control, nurse support, psychological interventions, improved communication, and greater access to specialist services rather than the isolated effect of the MDT.

### 6.5. Healthcare Utilization and Economic Impact

The increasing global prevalence of IBD highlights the need for sustainable, accessible, and cost-effective models of care [64]. In high-income regions, direct healthcare costs for IBD—including outpatient care, hospitalizations, and medications—have been reported at approximately US$9000–US$12,000 per person annually [65]. Healthcare systems face the dual challenge of achieving long-term remission while ensuring financial sustainability and equitable access to high-quality care [66]. In this context, the economic rationale for multidisciplinary care is based primarily on the possibility of reducing avoidable healthcare utilization through proactive monitoring, early treatment optimization, rapid-access pathways, and coordinated management of complex patients. Emergency department (ED) presentations and hospital admissions are relevant drivers of healthcare costs in IBD and represent an important indicator of disease control. Following MDT implementation, a survey reported fewer hospitalizations with an absolute reduction of 18 percentage points (48% vs. 30%, *p* = 0.02) [48]. Conversely, integrating the IBD MDT within the ED setting has been shown to improve the management of IBD-related emergencies. A virtual care program linking patients discharged from the ED with specialized IBD nurses was associated with a 45.7% reduction in subsequent ED visits compared with non-participants (*p* = 0.007) [67]. Furthermore, MDT interventions applied after ED discharge significantly reduced the subsequent ED access rate for at least 6 months [68]. These models may be particularly effective when they combine early nurse-led contact, symptom assessment, biomarker review, treatment optimization, and rapid escalation to gastroenterological or surgical review when required.

In an Australian tertiary IBD center, a 12-month evaluation of specialist nurse activity estimated that 27 hospital admissions, 171 inpatient bed-days, and 32 emergency department presentations were avoided. These are estimates of healthcare use avoided, rather than directly observed cost reductions [69]. In a separate Australian tertiary IBD service, a retrospective analysis of nurse encounters from October 2020 to September 2021 estimated net annual hospital savings of up to AUD $570,837.95 after deducting nursing salaries; the authors reported no other significant costs of running the service [70]. In a retrospective study reported as a conference abstract, a joint gastroenterology–rheumatology–pharmacy care model was associated with fewer consultations and laboratory tests than in the preceding year; ED visits (−73.1%) and hospital admissions (−50%) also decreased, but these differences were not statistically significant. The authors estimated annual savings of €47,220 across the 90 patients included [71].

These studies suggest possible reductions in healthcare use and context-specific savings, but they do not establish the cost-effectiveness of MDT care. Estimates of avoided visits, net savings, and clinical outcomes are not interchangeable, and program costs are often incompletely reported. Prospective economic evaluations need to compare the full costs and patient-relevant outcomes of defined MDT pathways with those of alternative care models.

## 7. Limitations and Challenges of MDT

Despite their potential benefits, integrated MDT models may also introduce organizational limitations [72]. They require additional resources and time for interprofessional meetings, communication and coordination, which may increase outpatient costs and, if poorly structured, risk delaying treatment decisions. A scheduled MDM should therefore not be a prerequisite for urgent management. In addition, recommendations may sometimes be formulated by specialists who have not personally assessed the patient; their input should consequently be based on complete and up-to-date clinical information, including comorbidities and patient preferences, and should complement rather than replace the assessment and clinical responsibility of the treating physician. MDT recommendations should be discussed with the patient before implementation. These potential limitations highlight the importance of clear referral pathways, defined responsibility for implementing decisions and regular auditing of the MDT process.

## 8. Implementation Strategies and Future Directions

**Figure 3:**
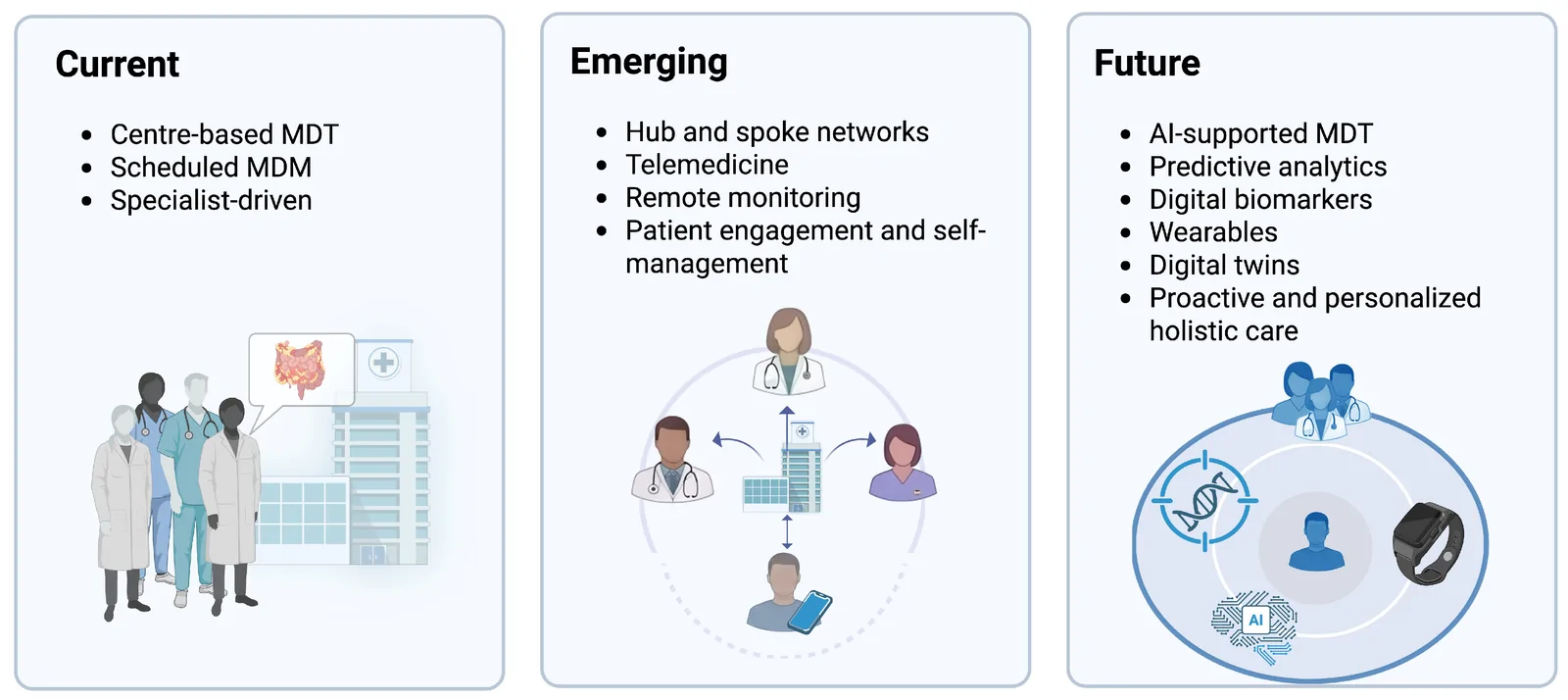
Evolution of the IBD MDT: current model to future integrated care. The model of multidisciplinary IBD care is evolving from traditional center-based and specialist-driven MDTs to interconnected care networks supported by telemedicine and remote monitoring, with future integration of artificial intelligence, digital biomarkers, wearables, and predictive tools with the potential to support more proactive and personalized care, subject to clinical validation. Abbreviations: AI = Artificial Intelligence; MDM = Multidisciplinary Meetings; MDT = Multidisciplinary Team. Created in BioRender. Teso, M. V. (2026) https://BioRender.com/wgtay0x.

Future developments in IBD management should aim to move care from a reactive and fragmented model towards integrated, proactive and patient-centered systems, including specialty medical homes and other comprehensive IBD care models [31,64]. A value-based approach may help assess these models by considering relevant outcomes, such as sustained remission and quality of life, alongside the resources required to achieve them [24,64]. To address workforce shortages and geographical disparities, hub-and-spoke networks could connect referral centers with peripheral hospitals, sharing specialist expertise while keeping appropriate care close to patients’ homes [73]. Telemedicine, remote monitoring, and structured referral pathways may support communication between centers and facilitate timely escalation of complex cases, particularly in rural or resource-limited settings [24,64,74]. Future MDTs should also become more participatory by systematically involving patients as active members in decision-making. Patient empowerment, structured self-management programs, and shared goal setting may improve treatment engagement and adherence (Figure 3).

Artificial intelligence (AI) may support, but should not replace, clinical judgment and shared decision-making within the MDT [75]. Potential applications include identifying patients who need review, endoscopic, histological, and radiological assessment, and prediction of disease progression or treatment response. In the near term, digital infrastructure may facilitate the sharing of clinical information across specialties. AI applications should be integrated into MDT workflows only after validation in the intended setting, with assessment of their clinical utility; their use cannot be assumed to resolve data fragmentation or improve patient outcomes [75,76]. Standardized data collection and digital literacy will also be needed to interpret outputs appropriately [53,77].

More advanced applications may include predictive models, digital biomarkers, and digital twins capable of integrating clinical, physiological, imaging, genomic, and proteomic information. These remain research directions rather than established components of IBD care. Wearable devices and remote-monitoring platforms may provide longitudinal data for research into flare prediction, but reliable prediction and prevention of flares have not been established in routine MDT care [74]. Clinical adoption will require external validation in diverse populations, reproducibility, and evidence of benefit to patients [76]. It will also require governance addressing privacy, bias, transparency, equity, and accountability frameworks [78]. Explainable and clinically interpretable AI may help build trust among both MDT members and patients, although explainability should be accompanied by prospective validation and evidence of clinical utility. Ultimately, technological innovation should remain subordinate to the principles of patient-centered care, shared decision-making, clinical safety, and equity of access [75,77].

## 9. Limitations

This review has several limitations. First, the studies discussed use heterogeneous definitions of multidisciplinary care and evaluate interventions ranging from formal case-discussion meetings to comprehensive service redesign. Their outcomes therefore cannot be interpreted as estimates of a single, uniform MDT effect. Second, much of the available evidence comes from observational, single-center or before-and-after studies, with variation in patient populations, comparators, follow-up periods and outcome definitions. Concurrent changes in staffing, access to specialist care, monitoring and treatment may confound associations between service implementation and outcomes. Finally, this narrative review did not use a systematic search and selection strategy or undertake a formal assessment of study quality or risk of bias. Relevant studies may therefore have been missed or selected unevenly, and the strength of the overall evidence cannot be graded formally. The findings should be interpreted as a critical narrative synthesis rather than a quantitative estimate of the effectiveness of MDT care.

## 10. Conclusions

The evidence reviewed encompasses both formal multidisciplinary case discussions and broader integrated IBD care pathways. Case discussions can influence diagnostic and treatment decisions, whereas associations with remission, quality of life, surgical outcomes, and healthcare utilization are often reported after implementation of more complex services. These outcomes cannot generally be attributed to multidisciplinary decision-making alone, because such services also involve specialist access, nursing support, structured monitoring, care coordination, and timely implementation of treatment plans.

However, MDT care remains inconsistently implemented across centers. Differences in team composition, access to specialist services, meeting frequency, referral criteria, documentation, workforce availability, and healthcare organization limit the comparability of existing models and contribute to geographical and socioeconomic inequities. Future development should focus on defining minimum standards for MDT composition and governance, establishing clear referral and communication pathways, protecting time for multidisciplinary activities, and implementing standardized indicators across clinical, patient-reported, surgical, organizational, and economic domains. Hub-and-spoke networks, nurse-led services, rapid-access clinics, telemedicine, and remote monitoring may help extend specialist expertise to peripheral and resource-limited settings while maintaining continuity of care.

## Data Availability

No new data were created or analyzed in this study. Data sharing is not applicable to this article.

## Abbreviations

The following abbreviations are used in this manuscript:

AI	Artificial intelligence
CD	Crohn’s disease
CS-free CR	Corticosteroid-free clinical remission
ECCO	European Crohn’s and Colitis Organisation
ED	Emergency department
EIM	Extraintestinal manifestation
FC	Flare clinic
GI	Gastrointestinal
IBD	Inflammatory bowel disease
IMID	Immune-mediated inflammatory disease
MDM	Multidisciplinary team meeting
MDT	Multidisciplinary team
N-ECCO	Nurses of the European Crohn’s and Colitis Organisation
PCP	Primary care provider
PRO	Patient-reported outcome
PROM	Patient-reported outcome measure
QoL	Quality of life
SIBDQ	Short Inflammatory Bowel Disease Questionnaire
SMH	Specialty Medical Home
SpA	Spondyloarthritis
TRAQ	Transition Readiness Assessment Questionnaire
UC	Ulcerative colitis
